# Association of Intestinal Barrier Damage and Mucosal Microbiota Dysbiosis with the TLR4/NF-κB Pathway in High-Salt Diet-Induced Hypertensive Rats

**DOI:** 10.3390/nu18203405

**Published:** 2026-10-17

**Authors:** Wenhao Si, Yao Chen, Yahui Ren, Kemin Li, Yixue Ma, Mengqi Liu, Hui Zheng, Yong Yang, Tao Zheng

**Affiliations:** School of Pharmacy, Hunan University of Chinese Medicine, Changsha 410208, China; kyoumachris613@gmail.com (W.S.); dawn821272@163.com (Y.C.); 15615351556@163.com (Y.R.); silan3796@gmail.com (K.L.); mayixue1030@163.com (Y.M.); liumengqi0227@outlook.com (M.L.); huizheng0104@163.com (H.Z.)

**Keywords:** high-salt diet, hypertension, intestinal mechanical barrier, gut mucosal microbiota, TLR4/NF-κB signal pathway

## Abstract

Background: A high-salt diet is a major risk factor for hypertension, but the role of the intestinal mucosal microbiota and the dysfunction of the intestinal barrier remain to be explored. Methods: Wistar rats were fed a normal diet (OF) and a high-salt diet (HSF) for 33 days to establish hypertension. Serum NO and TNF-α levels were quantified by enzyme-linked immunosorbent assay (ELISA). Histopathological changes in the intestine and vasculature were evaluated by hematoxylin and eosin (HE) staining. Immunofluorescence staining was employed to further assess intestinal barrier damage by detecting the expression levels of the tight junction proteins Claudin-1, Occludin, and ZO-1. Results: Mucosal microbiota was analyzed via 16S rRNA sequencing. TLR4/NF-κB pathway activation was analyzed by RT-qPCR and Western blot (WB). HSF rats developed hypertension with significant intestinal barrier damage, characterized by villus autolysis and reduced Claudin-1 and ZO-1 expression. High-salt intake significantly inhibited the growth of beneficial bacteria including *Brevibacterium* sp. RHS-str. 520 and *Levilactobacillus brevis*, while promoting harmful species such as *Streptococcus mitis* and *Nodosilinea nodulosa*. Correlation analyses showed that the species-level key differential bacteria including *Levilactobacillus brevis* and *Eubacterium tenue* were negatively correlated with both intestinal TNF-α protein expression and blood TNF-α levels. Mechanistically, a high-salt diet activated the intestinal TLR4/NF-κB pathway, upregulating TLR4, MyD88, *p*-P65, and TNF-α protein expression (*p* < 0.05). Conclusions: Collectively, these findings suggest that a high-salt diet may affect the intestinal mucosal microbiota, trigger TLR4-mediated intestinal inflammation, and impair the mucosal barrier, ultimately driving hypertension via an intestinal mucosal barrier–inflammation–blood pressure axis.

## 1. Introduction

Diet represents a lifelong modifiable risk factor for human health. Influenced by shifting dietary habits and lifestyles, processed foods and takeout meals have increasingly permeated daily life, propelling many countries to become high-salt-consuming nations. Data from the World Health Organization (2023) reveal that average salt intake remains markedly above recommended levels across diverse geographic regions, i.e., 8.9 g/day in the United States, 11.1 g/day (equivalent to 4357 mg of sodium) in Poland, and 7.1 g/day in the United Kingdom, despite targeted reduction efforts in the latter [1]. In China, the *Chinese Dietary Guidelines for Residents* (2022) recommend an adult salt intake of 5 g/day. Yet empirical surveys consistently show that average daily sodium consumption in multiple provinces ranges from 9.14 to 11.22 g, nearly doubling the guideline recommendation [2,3,4]. The above data indicate that a high-salt diet has become the predominant dietary habit in most parts of the world, highlighting that excessive salt intake is a public health issue that requires immediate and sustained intervention. Hypertension is one of the most direct and harmful diseases associated with high-salt diets. Classified by the International Classification of Diseases as a systemic chronic inflammatory disorder, it now affects over 1 billion people worldwide. According to six rounds of surveys, in 2018, China had 274 million adults aged 18–69 with hypertension, representing a standardized prevalence rate of 24.7% [5]. An epidemiological study involving 18 countries and over 100,000 participants revealed a significant association between daily salt intake and blood pressure and low-salt diets, demonstrating therapeutic effects on hypertension [6,7]. Quantitatively, when sodium excretion exceeds 5 g/day, systolic blood pressure (SBP) increased by 2.58 mmHg per gram of sodium excreted; for sodium excretion between 3 and 5 g/day, SBP increased by 1.74 mmHg per gram of sodium excreted; and for sodium excretion <3 g/day, SBP increased by 0.74 mmHg per gram of sodium excreted [7]. Therefore, paying attention to the impact of salt intake on blood pressure is of vital importance for reducing the incidence and mortality rate of hypertension and its related complications.

The intestinal mucosal immune system is the largest immune organ in vertebrates, and the intestinal mucosa serves as the primary site for salt absorption in the body. The intestinal barrier consists of mechanical, chemical, microbial, and immune barriers, which are closely linked to the gut microbiota. The mechanical barrier is primarily composed of epithelial cells and intercellular tight junctions. Tight junctions, which include Claudin-1, Occludin, zonula occludens-1 (ZO-1), and junctional adhesion molecules, are the key structures responsible for the barrier function of the mechanical barrier [8]. The expression level of tight junction proteins directly reflects the integrity of the intestinal barrier. Tight junction proteins in the gastrointestinal tract enable the transport of sodium ions through intercellular pathways. The chemical barrier is primarily composed of mucins. Mucins, specialised glycoproteins produced by goblet cells, form a major component of the chemical barrier in mice and humans. These mucins collectively contribute to regulating host–environment interactions at mucosal surfaces through myriad mechanisms ranging from forming physiochemical barriers to regulating signal transduction pathways in epithelial cells [9,10]. The gut microbiota—comprising both luminal and mucosal populations—is a key component of the intestinal barrier and plays a vital role in maintaining its structural and functional integrity [11]. Disruption of this barrier can trigger local or systemic immune responses, a critical pathogenic mechanism in chronic inflammatory diseases. Damaged intestinal barrier function promotes endothelial dysfunction and vascular stiffening, thereby activating pathological vascular inflammation [12]. Concurrently, bacteria and their metabolites may enter the circulation, further amplifying systemic inflammatory responses. Research indicated that both exogenous and endogenous factors could alter gut microbiota composition. Dietary intake or medication influences gut microbial composition. Under prolonged high-salt dietary patterns, damaged intestinal barrier function facilitates bacterial translocation, allowing gut bacterial products to enter the systemic circulation, which activates systemic inflammation and triggers hypertension [13]. Dysbiosis of the gut microbiota may be associated with the pathogenesis of hypertension [14,15]. Therefore, the integrity of the intestinal barrier function is closely related to the pathogenesis of high-salt-induced hypertension.

Toll-like receptor 4 (TLR4) serves as a critical molecular bridge between inflammatory responses and metabolic homeostasis, and has thus been recognized as a promising therapeutic target for metabolic disorders. TLR4 expression is tightly linked to the intestinal mucosal microbiota, with which it maintains a dynamic equilibrium under physiological conditions. In pathological states, however, the composition of the intestinal mucosal microbiota undergoes significant changes, accompanied by corresponding alterations in intestinal TLR4 expression [16]. The TLR4/MyD88/NF-κB pathway mediates various chronic diseases [17]. Inflammation (including elevated levels of pro-inflammatory factors such as TNF-α and IL-6) and immune pathways play a significant role in the deterioration of hypertension [18]. This study established a rat model of hypertension via a high-salt diet. By examining parameters related to intestinal mucosal microbiota, intestinal mucosal barrier function, protein expression changes in the TLR4/NF-κB pathway on the intestinal mucosa, and blood parameters in hypertensive rats, we aimed to preliminarily investigate the potential roles of gut microbiota and intestinal mucosal barrier in the pathogenesis of salt-induced hypertension, and to explore the possible mechanisms by which high-salt-induced intestinal barrier damage contributes to the development of hypertension, thereby providing an experimental basis for future research.

## 2. Materials and Methods

### 2.1. Experimental Procedure

**Figure 1:**
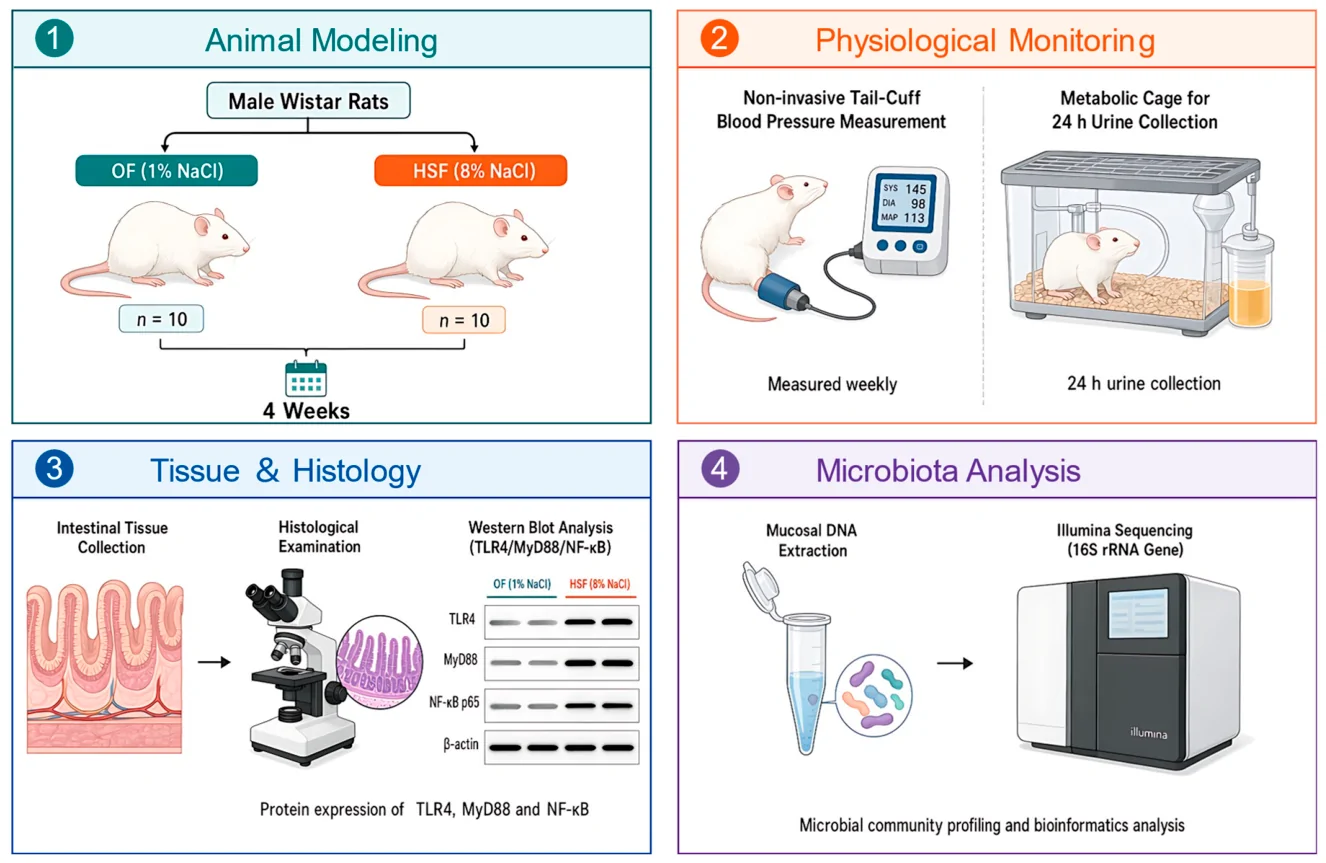
Experimental Flowchart.

The study protocol was finalized before research initiation and not prospectively registered. A workflow flowchart is shown in Figure 1. Hypertensive rat models were induced by a high-salt diet. After model construction, intestinal barrier-related indicators were evaluated, and TLR4/NF-κB signaling pathways in intestinal mucosa were determined. Meanwhile, intestinal mucosal microbiota composition was analyzed.

### 2.2. Reagents and Instruments

Sodium chloride, paraformaldehyde, sodium pentobarbital, anhydrous ethanol, neutral gum (Sinopharm Chemical Reagent Co., Ltd., Shanghai, China). Pierce™ Rapid Gold BCA Protein Assay Kit, 5× Protein Loading Buffer, AP, RIPA Lysis Buffer, Phosphoprotein Inhibitor, TRIS, Glycine, SDS, Tween 20, ECL Chemiluminescent Substrate (Wuhan Pinofei Bio, Wuhan, China, Cat. No. P0009, PN0033, P0167, P0005, P0007, PN0034, P0098, P0017, P0164, PN0021). Phosphorylated protease inhibitor, protease inhibitor (Beyotime, Shanghai, China, Cat. No. P1082, P1006). Medical X-ray film (Kodak, Rochester, NY, USA, Catalog No. XBT-1). Protein Marker (Biosafe, Shanghai, China, Catalog No. P8028L). TLR4, MyD88, TNF-α antibodies (Proteintech, Rosemont, IL, USA, Catalog Nos. 66350-1-IG, 67969-1-IG, 60291-1-IG). *p*-P65 antibody (Abways, Shanghai, China, catalog no. cy6367). IL-6 antibody (HUABIO, Hangzhou, China, catalog no. R1412-2). β-actin antibody (Servicebio, Wuhan, China, catalog no. GB11001). Secondary antibodies: Goat anti-Rabbit IgG (H + L), Goat anti-mouse IgG (H + L) (Servicebio, Wuhan, China, Cat. No. G1213, G1214). Nitric Oxide Detection Kit (Biyuntian Bio, Shanghai, China, Cat. No. S0021S). Rat TNF-α Enzyme-Linked Immunosorbent Assay Kit (Xinbosheng Bio, Shenzhen, China, Cat. No. E RC102a.96).

Rat non-invasive tail artery blood pressure monitor (Softron Biotechnology, Beijing, China, Model BP-2010A). High-speed tissue grinder (Servicebio, Wuhan, China, Model KZ-II). High-Speed Microcentrifuge with Freezing Function (SCILOGEX, Berlin, CT, USA, Model CF1524R). Multifunctional Microplate Reader (BIOBASE, Jinan, China, Model BK-EL10C). Chemiluminescence Imager (Hangzhou Shenhua Technology Co., Ltd., Hangzhou, China, Model SH-compact523).

### 2.3. Experimental Animals and Feed

#### 2.3.1. Animals

A total of 20 SPF-grade Wistar rats, 6 weeks old, male, weighing 150 ± 10 g, were purchased from Beijing Huafukang Bioscience Co., Ltd. (Beijing, China) (Certificate No. ZS-202410300010). All animals were housed at the Hunan University of Chinese Medicine Laboratory Animal Center under environmental conditions of 23–25 °C room temperature, 50–70% relative humidity, a 12-h light/dark cycle, and ad libitum access to water and food. Rats were euthanized with a 4% sodium pentobarbital solution prior to tissue collection. Animal experiments were reviewed and approved by the Laboratory Animal Ethics Committee of Hunan University of Chinese Medicine (Approval No. SLBH-202407040002). All experimental procedures and animal care were performed in compliance with the ARRIVE guidelines 2.0 (Animal Research: Reporting of In Vivo Experiments). All experimental manipulations were performed in accordance with the 3R guidelines (replacement, reduction, and refinement), striving to minimize animal usage and reduce distress. Rats were excluded if they (1) exhibited signs of illness or a body weight loss exceeding 20% during the one-week acclimation period; (2) reached humane endpoints during high-salt modeling, such as severe weight loss, impaired mobility, or moribund condition, and were euthanized prematurely; (3) died unexpectedly before the scheduled sampling time point, preventing the collection of samples required for subsequent measurements; or (4) failed to develop a hypertension phenotype following high-salt diet intervention and were therefore classified as salt-resistant individuals and excluded. Both the normal control group and the high-salt hypertension group in this study underwent animal exclusion and euthanasia procedures in accordance with the above criteria. Four rats were excluded from the high-salt diet group because they exhibited salt resistance and failed to develop stable hypertension.

#### 2.3.2. Feed

The maintenance diet for rats was purchased from Tianjin Ke’ao Xie Li Feed Co., Ltd. (Tianjin, China). As shown in Table 1, the standard nutritional composition was: moisture < 10%, crude protein ≥ 18%, crude fat ≥ 4%, crude fiber ≤ 5%, crude ash ≤ 8%, calcium 1.0–1.8%, phosphorus 0.6–1.2%. High-salt feed was purchased from Tianjin Ke’ao Xie Li Feed Co., Ltd. As Table 1 shows, the standard feed contained 1% sodium chloride (NaCl). The high-salt feed was formulated by adding NaCl to the standard feed at 8% of the total weight. Both feeds had identical energy supply ratios.

### 2.4. Experimental Methods

#### 2.4.1. Animal Grouping and Modeling

A total of 20 Wistar rats were randomized into two groups (*n* = 10 per group) after three days of adaptive feeding. Random grouping of experimental rats was performed using the random number-generation function in Microsoft Excel. Rats were randomly divided into a normal control group (abbreviated as OF group) and a high-salt-induced hypertension group (abbreviated as HSF group). The OF group was fed standard chow, while the HSF group was fed an 8% high-salt diet to establish the high-salt-induced hypertension animal model. Rats were housed two per cage, resulting in five cages for each experimental group. To eliminate confounding factors, cage positions on the rack were horizontally rotated every three days to minimize positional biases arising from fixed placement, such as variations in noise, light intensity, and ventilation. Body weight and food intake were recorded weekly, and 24-h urine output was measured using metabolic cages. Ten rats were initially assigned to each group at the start of the experiment, and six rats per group remained after excluding salt-resistant rats from the high-salt diet group. For biochemical and histological assays, these six animals from each group served as the analysis set to ensure technical consistency. The sample size (*n* = 6 per group) in this study was basically consistent with that adopted in our previous studies on high-salt-induced hypertension and comparable published studies.

#### 2.4.2. Blood Pressure Measurement and Hypertension Model

SBP and DBP were measured in each rat using the tail cuff volumetric recording technique at 8:00 AM every Tuesday and Friday. Each rat underwent three repeated measurements, and the mean value was recorded. Compared to the control group, rats in the experimental group were determined to have salt-induced hypertension when the increase in DBP exceeded 5–10%, the increase in SBP exceeded 10–15% or both SBP and DBP significantly increased [19].

To avoid operator-dependent variability, partial blinding was implemented in this study, as follows: The first author Wenhao Si and corresponding author Tao Zheng were unblinded and had access to group allocation throughout group assignment, experimental procedures, outcome assessment, and data analysis. In contrast, the remaining technical staff performing experimental operations were kept blinded to group information to minimize assessment bias.

#### 2.4.3. Biochemical Analysis and Organ Index Measurement

Rats from the OF and HSF groups were euthanized by intraperitoneal injection of sodium pentobarbital (45 mg/kg), and blood was immediately collected via the abdominal aorta into sterile tubes. After standing for 1 h at 4 °C, serum was obtained by centrifugation at 4000 rpm for 15 min. Serum levels of TNF-α and NO were measured using ELISA kits.

At the conclusion of the experiment, all rats were weighed and euthanized. The major organs, including the liver, spleen, and kidneys, were rapidly excised, carefully cleaned of adherent adipose tissue and fascia, and blotted dry with filter paper. Organ indices were then calculated as follows: spleen index (%) = spleen weight (g)/rat body weight (g) × 100%; kidney index (%) = kidney weight (g)/rat body weight (g) × 100%; liver index (%) = liver weight (g)/rat body weight (g) × 100%.

#### 2.4.4. HE Staining and Intestinal Morphology Examination

Under sterile conditions, intestinal segments (jejunum to ileum segments) approximately 3 cm in length and blood vessels were collected from each group of rats and fixed in 4% paraformaldehyde. The specimens were dehydrated with ethanol and xylene, embedded in paraffin, sectioned into 4 μm thick slices, and examined under a light microscope for pathological changes using HE staining. ImageJ Pro Plus 6.0 image analysis software was used to measure villus height (VH), crypt depth (CD), epithelial length (EL), and goblet cell count in rat intestines, and calculate the villus-to-crypt ratio (V/C) and the number of goblet cells per unit length. Five random measurements per sample were averaged.

#### 2.4.5. Detection of Claudin 1, Occludin, and ZO-1 Protein Expression Using Immunofluorescence Staining

Paraffin-embedded tissue sections were deparaffinized and rehydrated, then incubated in 3% hydrogen peroxide in deionized water for 10 min. Sections were rinsed three times with PBS for 2 min each. Primary antibodies against Claudin-1 (1:200 dilution), Occludin (1:200 dilution), and ZO-1 (1:200 dilution) were added separately and incubated overnight at 4 °C. Sections were washed with PBS. Polymer Helper was then added and incubated at room temperature for 20 min, followed by PBS washing. Sections were incubated with fluorophore-conjugated secondary antibodies (Alexa Fluor 488/594). Nuclei were counterstained with DAPI. The stained sections were observed using immunofluorescence microscopy, and the fluorescence intensity was quantified using ImageJ software.

#### 2.4.6. WB Analysis

Rats were euthanized, and the jejunum to ileum segments were extracted. WB analysis was performed to determine the relative expression levels of TLR4, MyD88, *p*-P65, IL-6, and TNF-α relative to β-actin. Proteins were separated by SDS-PAGE electrophoresis, transferred to PVDF membranes, and blocked with 5% skim milk in a decolorization shaker for 1 h. Primary antibodies were incubated overnight at 4 °C, followed by secondary antibody incubation for 1 h. Luminescence detection was performed using an ECL mixture.

#### 2.4.7. RT-qPCR Analysis

mRNA was extracted from rat jejunal and ileal segments and reverse-transcribed into cDNA. The relative expression levels of mRNA were quantified using SYBR Green according to the instructions of the quantitative PCR kit. Specific primers were used, with the β-actin gene serving as an internal control, and the relative mRNA levels of the target gene were calculated using the 2^−ΔΔCt^ method.

Using specific primers, which were synthesized by Sangon Biotech Co., Ltd. (Shanghai, China): β-actin, 5′-CCCGCGAGTACAACCTTCTTG-3′(F), 5′-GTCATCCATGGCGAACTGGTG-3′(R); IL-6, 5′-AAGTCCGGAGAGGAGACTTCA-3′(F), 5′-GAATTGCCATTGCACAACTCTTTTC-3′(R); TNF-α, 5′-CCACGTCGTAGCAAACCAC-3′(F), 5′-CCACCAGTTGGTTGTCTTTGAG-3′(R); P65, 5′-ACCTGCTGCTTTTGTGTCTG-3′(F), 5′-TCATCCTTAAGGGCCTGATCC-3′(R); MyD88, 5′-GCAGTTTGTTGGATGCCTGG-3′(F), 5′-TTCTGGCAGTCCTCCTCGAT-3′(R); TLR4, 5′-GATCTGAGCTTCAACCCCCTG-3′(F), 5′-TCAATTGTCTCAATTTCACACCTGG-3′(R).

#### 2.4.8. Intestinal Mucosal Microbiota Analysis

Immediately after euthanizing rats, place them in a sterile environment. Harvest the jejunal and ileal segments as the test specimens, expel the chyme contents, incise the intestine, and wash the intestinal wall contents with pre-boiled and cooled sterile saline. Blot dry with sterile filter paper. Scrape the intestinal mucosa with a coverslip and place it into a sterile centrifuge tube containing 2 times the intestinal weight in saline for subsequent analysis. Microorganisms on the intestinal mucosa were extracted and detected. The full-length bacterial 16S rRNA gene was amplified using specific primer pairs, with the forward primer sequence AGAGTTTGATCMTGGCTCAG and the reverse primer sequence ACCTTGTTACGACTT. PCR amplification, purification, and recovery were performed using Illumina’s TruSeq Nano DNA LT Library Prep Kit to prepare sequencing libraries. Library quality control was conducted on an Agilent Bioanalyzer. Libraries were quantified using the Quant-iT PicoGreen dsDNA Assay Kit on the Promega QuantiFluor fluorescence quantification system. Double-end sequencing was performed using the NovaSeq sequencer (NovaSeq 6000 SP Reagent Kit, 500 cycles; Illumina, San Diego, CA, USA). All samples achieved an average sequencing depth of 50,000 reads per sample. Taxonomic annotation of microbial sequences was performed based on the NCBI nt database (Version 1.1). In this study, rats from the OF group were set as the negative control for microbiome sequencing analysis.

Microbiome bioinformatics were performed with QIIME2 2022.11, with slight modifications according to the official tutorials (https://docs.qiime2.org/2022.11/tutorials/, accessed on 31 January 2025). Briefly, raw sequence data were demultiplexed using the demux plugin followed by primer cutting with the cutadapt plugin. Sequences were then quality-filtered, denoised, and merged, and chimeric sequences were removed using the DADA2 plugin. Sequence data analyses were mainly performed using QIIME2 and R packages. ASV-level alpha diversity indices, including Chao1 richness estimator, Shannon diversity index, Simpson index, and Faith’s PD, were calculated using the ASV table in QIIME2. Beta diversity analysis was performed to investigate the structural variation in microbial communities across samples using Jaccard metrics. Principal component analysis (PCA) was also conducted based on the genus-level compositional profiles. Nonmetric multidimensional scaling (NMDS) was also performed to evaluate the variation in microbial community structure among samples. A Venn diagram was generated to visualize the shared and unique ASVs among groups using the R package version 3.6.0 “VennDiagram”, based on the occurrence of ASVs across groups regardless of their relative abundance. LEfSe (Linear discriminant analysis effect size) was performed to identify differentially abundant taxa between groups with an LDA threshold of 3.0. A random forest model was constructed using the R package randomForest to identify taxa associated with hypertension. The input variables were the relative abundances of differential taxa at the species level, and the outcome was group assignment (OF vs. HSF). The model was evaluated using ROC curves, and variable importance was assessed by mean decrease in accuracy, and the top 20 taxa with the highest importance scores were selected as candidate biomarkers.

#### 2.4.9. Data Analysis

All statistical analyses were performed using SPSS 22.0, and a two-sided *p* value < 0.05 was considered statistically significant. Normality of all continuous variables was assessed using the Shapiro–Wilk test, and data were presented as mean ± standard deviation (SD) for normally distributed variables. Between-group comparisons were performed using independent-samples *t*-tests, with Welch’s *t*-test applied when equal variances could not be assumed. One-way ANOVA followed by Tukey’s post hoc test was used for comparisons involving multiple morphometric parameters, and the Mann–Whitney U test was used for non-normally distributed data. For variables measured repeatedly over time (weekly blood pressure, body weight, food intake, and 24-h urine volume), repeated-measures two-way ANOVA (group × time) followed by Bonferroni’s post hoc test was used. RT-qPCR data were normalized to β-actin as the internal reference gene and analyzed using the 2^−ΔΔCt^ method. Analyses of microbiota alpha and beta diversity and LEfSe were performed as described in Section 2.4.8. Spearman’s correlation analysis was performed to clarify the correlation between blood biochemical indicators and intestinal mucosal microbiota, and the results were visualized using a heatmap. Bar charts were generated using GraphPad Prism 10.1.2. The exact number of rats (n) used for each analysis is specified in the corresponding figure and table legends.

## 3. Results

### 3.1. High-Salt Diet Induces Hypertension in Rats

As shown in Table 2, compared with the OF group, the HSF rats exhibited a gradual increase in SBP and DBP starting from day 2 to day 23 of the high-salt diet. By day 5, both SBP and DBP in the HSF group were significantly higher than those in the OF group (*p* < 0.01, *p* < 0.05). However, by day 12, no significant difference was observed (*p* > 0.05), indicating that rats under high-salt diet stress exhibited significant blood pressure fluctuations. By days 26 to 30 of high-salt feeding, the HSF group blood pressure was markedly higher than the OF group blood pressure (*p* < 0.001), confirming that the rats had developed stable hypertension.

### 3.2. Effects of High-Salt Diet on Food Intake and Body Weight in Rats

**Figure 2:**
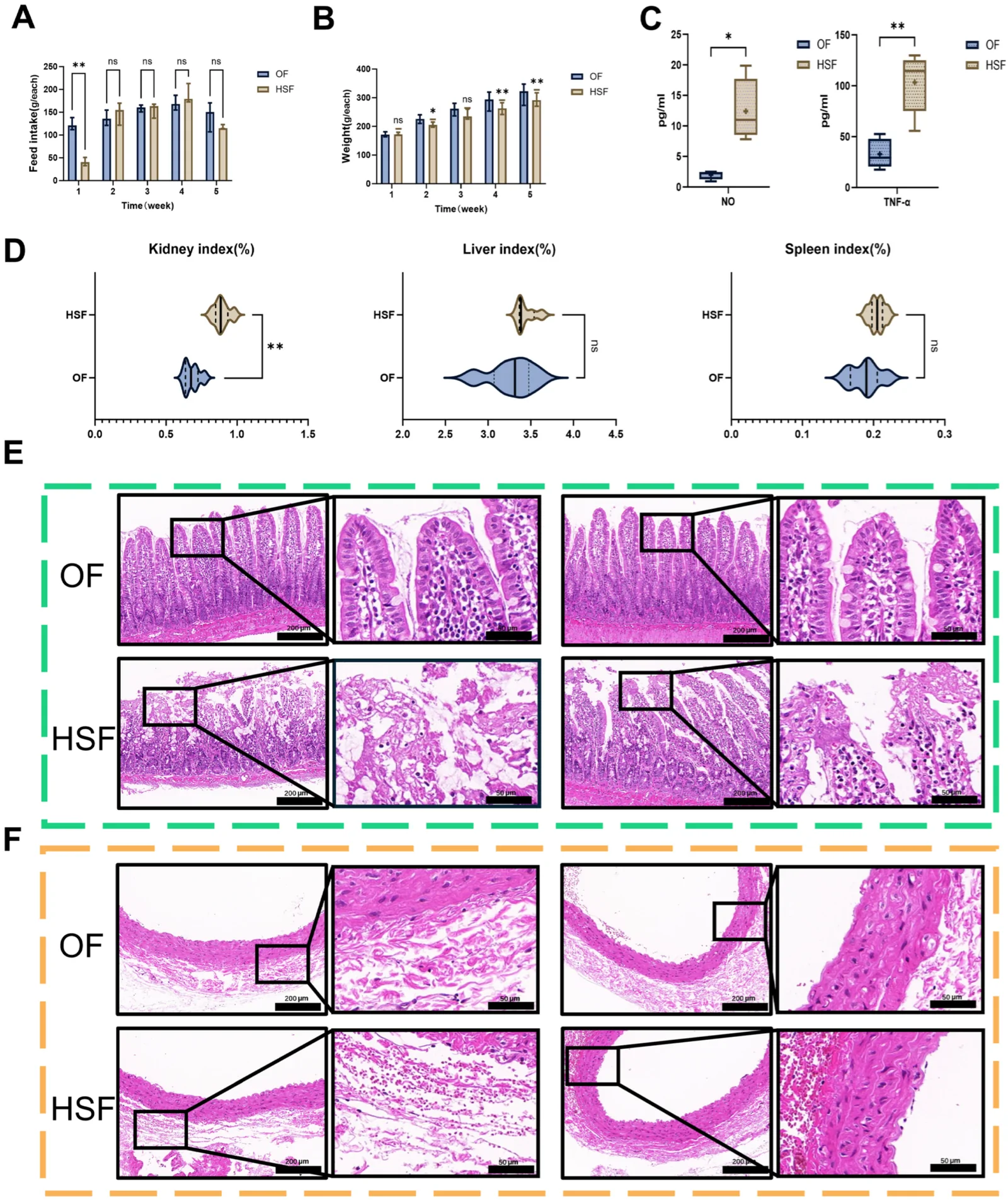
Blood pressure, blood parameters, organ indices, and HE staining results in rats. Food intake ($\bar{x}$ ± s, *n* = 6) (**A**); body weight ($\bar{x}$ ± s, *n* = 6) (**B**); serum biochemical test results ($\bar{x}$ ± s, *n* = 6) (**C**); comparison of organ indices for rat kidney, liver, and spleen ($\bar{x}$ ± s, *n* = 6) (**D**); HE staining results (intestinal tissue) (**E**); HE staining results (vascular tissue) (**F**); * indicates *p* ≤ 0.05, ** indicates *p* ≤ 0.01, ns indicates not significant.

As shown in Figure 2A, rats in the HSF group showed fluctuations in food intake. During the first week of high-salt diet administration, their food consumption was significantly lower than that of the OF group (mean difference = 81.85, 95% CI: 51.87 to 111.8, *p* = 0.023). However, starting from the second week, their intake recovered to levels comparable to the OF group, with no significant difference observed (*p* > 0.05). By comprehensively analyzing the feeding conditions and weight data, it was found that after high-salt diet intake, the weights of the OF group and the HSF group fluctuated during weeks 1–3. However, starting from the fourth week, the weights of the rats in the high-salt diet group were significantly lower than those in the hypertension group (mean difference = 29.49, 95% CI: 9.271 to 49.70, *p* = 0.0012), indicating that high-salt diet intake in the later stage does not affect the rats’ appetite and significantly reduces the rats’ weight, while also causing an increase in blood pressure (Figure 2B).

### 3.3. Increased Blood TNF-α and NO Levels

As shown in Figure 2C, serum TNF-α concentrations in the OF and HSF groups were 32.77 pg/mL and 103.303 pg/mL, respectively. The HSF group had significantly higher TNF-α levels than the OF group (mean difference = 70.53, 95% CI: 40.02 to 101.0, *p* = 0.0009). Regarding serum NO content, the content in the HSF group (12.41 pg/mL) was significantly higher than that in the OF group (1.98 pg/mL) (mean difference = 10.43, 95% CI: 2.260 to 18.60, *p* = 0.027).

### 3.4. Effects on Kidney, Liver, and Spleen Indices

As shown in Figure 2D, the high-salt diet significantly increased kidney mass in rats. The kidney index in the HSF group was significantly higher than that in the OF group (mean difference = 0.2083, 95% CI: 0.1388 to 0.2779, *p* < 0.0001). No significant differences were observed in liver and spleen indices between the two groups (*p* > 0.05), indicating that high-salt diet intake primarily affects rat kidneys.

### 3.5. Intestinal and Vascular HE Staining Results

As shown in Figure 2E, intestinal HE staining revealed that the mucosal layer of the OF group exhibited abundant, well-arranged intestinal villi with no significant epithelial cell detachment. The lamina propria contained small intestinal glands with regular morphology and compact arrangement. The submucosa displayed fine connective tissue, while the muscularis layer showed no obvious abnormalities in smooth muscle cells and no significant inflammatory cell infiltration. In the HSF group, intestinal villi exhibited apical autolysis and incomplete mucosal architecture, indicating damaged intestinal barrier. Vascular HE staining revealed that the OF group exhibited well-defined vascular architecture and the internal elastic lamina displayed clear, wavy structures. The loose connective tissue of the adventitia showed no significant abnormalities or inflammatory cell infiltration. However, in the HSF group, there was localized extravasation of red blood cells in the adventitia of the arteries, which reflects that the high-salt diet-induced hypertension in rats also led to vascular damage (Figure 2F).

### 3.6. High-Salt Diet Induced Intestinal Barrier Damage in Rats

**Figure 3:**
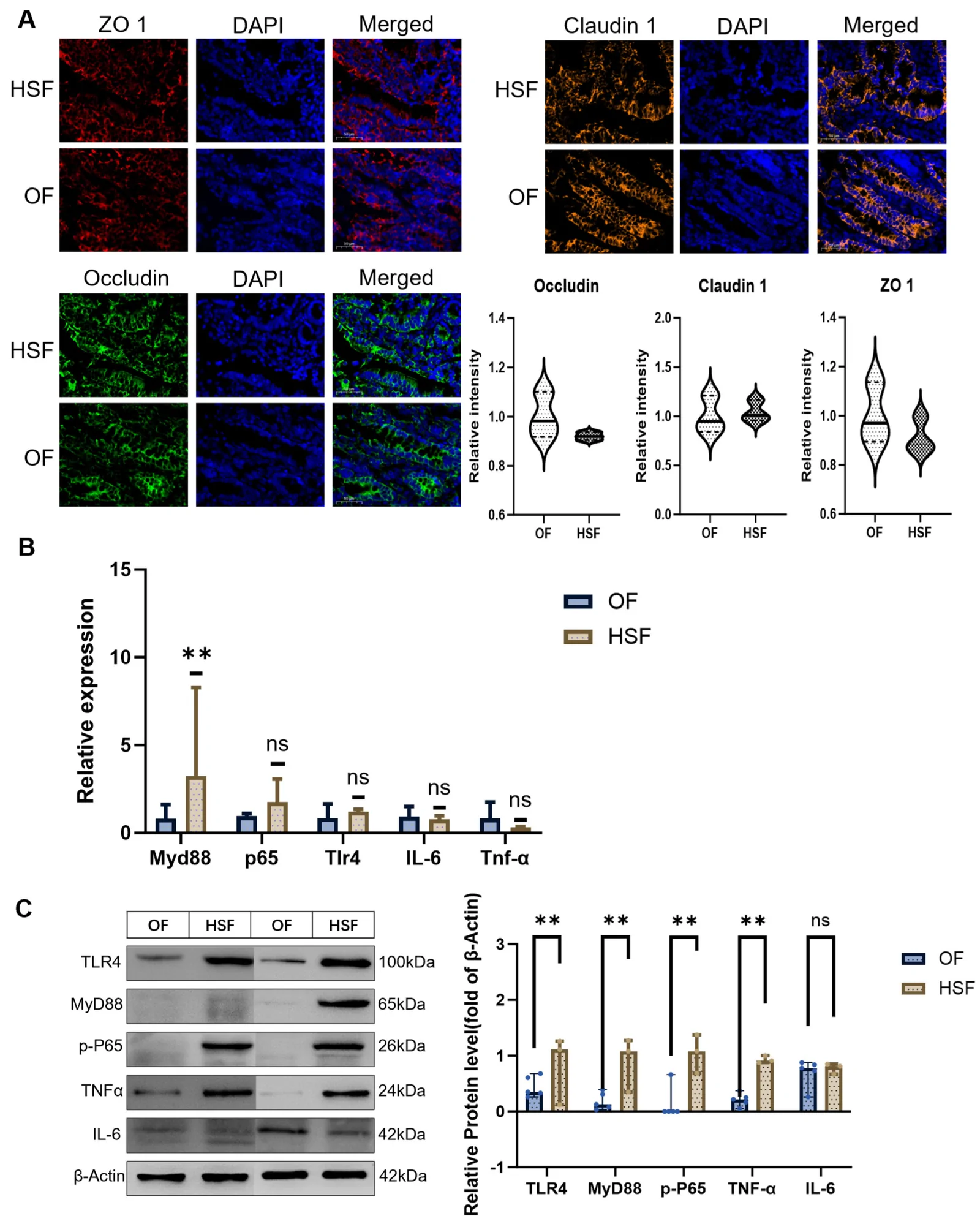
Results of fluorescence staining, RT-qPCR, and WB of rat intestinal tissue (*n* = 6). Fluorescent staining and grayscale analysis of tight junction proteins in rat intestines (**A**); RT-qPCR results in rat intestinal tissue (**B**); WB results in rat intestinal tissue (**C**); ** indicates *p* ≤ 0.01, ns indicates not significant.

Table 3 shows reduced VH, shallower CD, decreased goblet cell number, and shorted EL in the HSF group. The EL in the HSF group was significantly lower than that in the OF group (mean difference = −0.1547, 95% CI: −0.2303 to −0.07914, *p* = 0.0003). Goblet cells per unit length was higher in the HSF group than in the OF group, but the difference was not statistically significant. The concurrent decrease in VH and EL indicates a significant reduction in mucosal surface area, suggesting that high salt intake impairs the regenerative capacity and homeostatic renewal of the intestinal epithelium. Furthermore, the reduction in goblet cells (the primary source of the mucus layer) implies impaired chemical barrier function [9,10]. In Figure 3A, the results of fluorescence staining of tight junction proteins in the rat intestine showed that although the expression levels of Occludin, Claudin-1 and ZO-1 proteins in the HSF group were not significantly different from those in the OF group (*p* > 0.05). Taken together with the significant morphological changes in VH, CD, and EL, these results suggest that a high-salt diet may exert a potential impact on the mechanical barrier of the rat intestine while inducing hypertension, although further experimental validation is required.

### 3.7. Effects of High-Salt Diet on the MyD88 Pathway in Rat Intestines

As shown in Figure 3B, the relative mRNA expression levels of MyD88, P65, and TLR4 in the intestines of the HSF group were higher than those in the OF group, with the relative expression of MyD88 being significantly higher than that in the OF group (mean difference = −3.776, 95% CI: −6.239 to −1.313, *p* = 0.0016). As shown in Figure 3C, the protein expression levels of TLR4 (mean difference = −0.7440, 95% CI: −1.214 to −0.2738, *p* = 0.0007), MyD88 (mean difference = −0.7120, 95% CI: −1.182 to −0.2418, *p* = 0.0013), *p*-P65 (mean difference = −0.9147, 95% CI: −1.385 to −0.4445, *p* < 0.0001), and TNF-α (mean difference = −0.7180, 95% CI: −1.188 to −0.2478, *p* = 0.0011) were significantly higher than the OF group. Although the protein expression of IL-6 was not significantly different between the two groups (*p* > 0.05), it was higher in the HSF group than in the OF group. This indicates that a high-salt diet induced changes in the expression of genes and proteins related to the MyD88 pathway in the rat intestine, thereby activating the pathway.

### 3.8. Effects of High-Salt Diet on Gut Mucosal Microbial in Rats

#### 3.8.1. Effects on Intestinal Mucosal Microbiota Diversity and Composition in Rats

**Figure 4:**
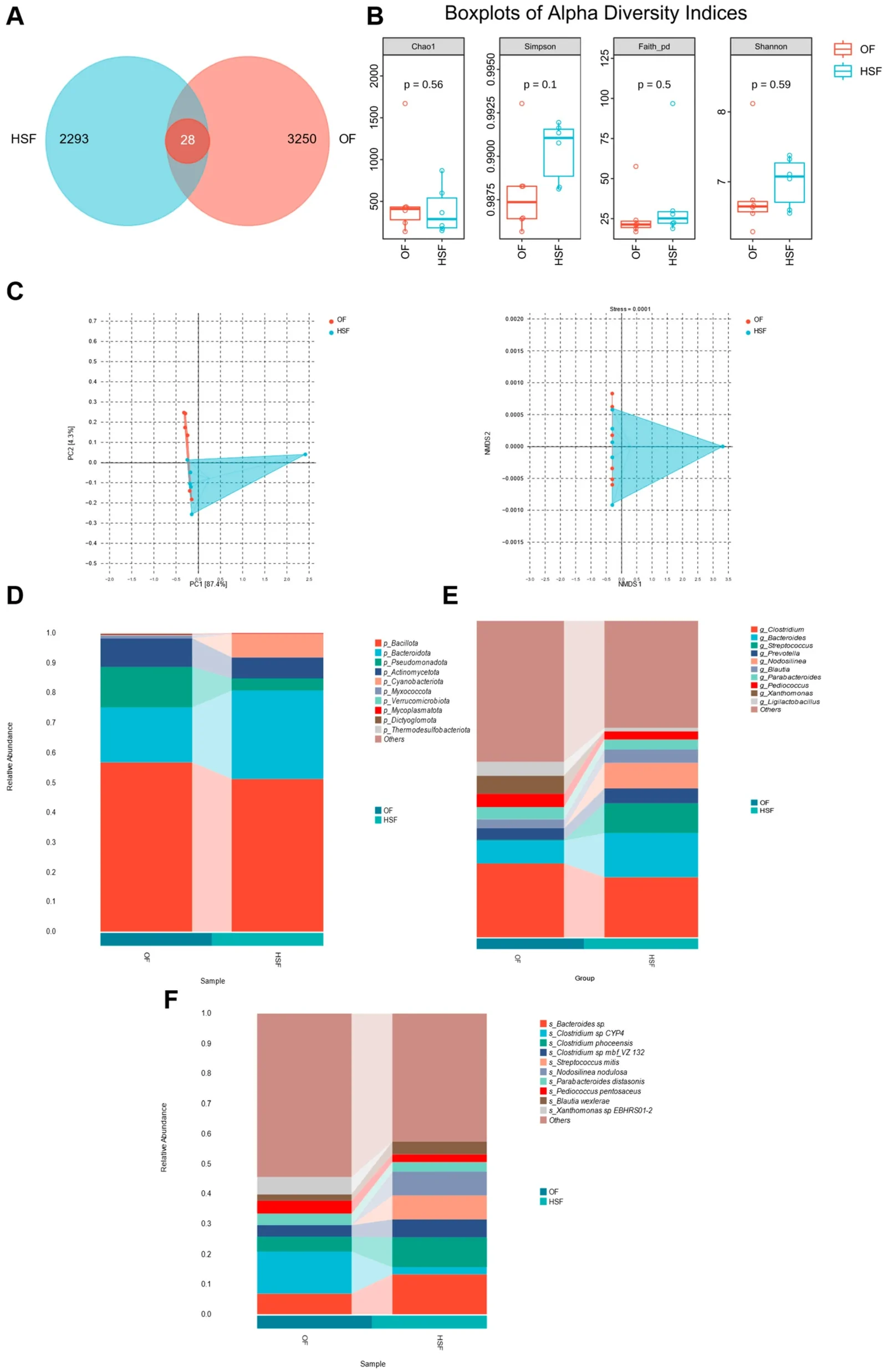
Analysis of gut microbiota species richness and composition (*n* = 6). Venn diagram showing shared and unique ASVs between OF and HSF groups (**A**); alpha and beta diversity analyses (**B**,**C**); relative abundance at phylum, genus, and species levels (**D**–**F**).

The ASVs of the OF group and the HSF group are shown in Figure 4A. The number of ASVs in the HSF group was lower than that in the OF group, being 2321 and 3278 respectively, and the number of shared ASVs between the two groups was 28. From the perspective of microbial diversity, the results of α-diversity and β-diversity for both groups showed that although there were no significant differences in richness and uniformity of the intestinal mucosal microbiota in the two groups of rats with a high-salt diet, there were differences in their distribution (Figure 4B,C). Figure 4D–F display the top 10 microbial phylum, genus, and species compositions in both groups. At the phylum level (Figure 4D), Bacillota, Bacteroidetes, Pseudomonadota, and Actinomycetota were common dominant phyla in the intestines of both OF and HSF rats. Among these, the relative abundances of Bacillota, Pseudomomadota, and Actinomycetota were higher in the OF group than in the HSF group. Cyanobacteriota was exclusively present in the HSF group. At the genus level (Figure 4E), the top 10 genera were *Clostridium*, *Bacteroides*, *Streptococcus*, *Prevotella*, *Nodosilinea*, *Blautia*, *Parabacteroides*, *Pediococcus*, *Xanthomonas*, and *Ligilactobacillus*. Among these, the abundance of *Clostridium* and *Xanthomonas* in the OF group was higher than that in the HSF group, while the abundance of *Bacteroides*, *Blautia*, *Streptococcus* were lower than that in the HSF group. Notably, *Blautia* and *Streptococcus* were significantly lower in the OF group than in the HSF group (*p* < 0.05). The genus *Nodosilinea*, belonging to the cyanobacteria phylum, was exclusively present in the HSF group and classified as a harmful bacterium [20]. *Xanthomonas* was exclusively present in the OF group. At the species level (Figure 4F), the top 10 dominant species were *Bacteroides* sp., *Clostridium* sp. CYP4, *Clostridium* phoceensis, *Clostridium* sp. mbf VZ 132, *Streptococcus mitis*, *Nodosilinea nodulosa*, *Parabacteroides distasonis*, *Pediococcus pentosaceus*, *Blautia wexlerae*, and *Xanthomonas* sp. EBHRSOI-2. Among these, the relative abundances of *Clostridium* sp. CYP4, *Parabacteroides distasonis*, *Pediococcus pentosaceus*, and *Xanthomonas* sp. EBHRSOI-2 were higher in the OF group than in the HSF group, while the relative abundances of *Bacteroides* sp. and *Clostridium phoceensis* were lower in the OF group. *Streptococcus mitis* and *Nodosilinea nodulosa* were present only in the HSF group. *Xanthomonas* sp. EBHRSOI-2 was present only in the OF group, and it acts as a potentially beneficial commensal [21]. Results at both the genus and species levels indicated that the high-salt diet suppressed the growth of beneficial gut mucosal bacteria, leading to gut mucosal microbiota dysbiosis.

#### 3.8.2. Gut Mucosal Characteristic Microbiota

**Figure 5:**
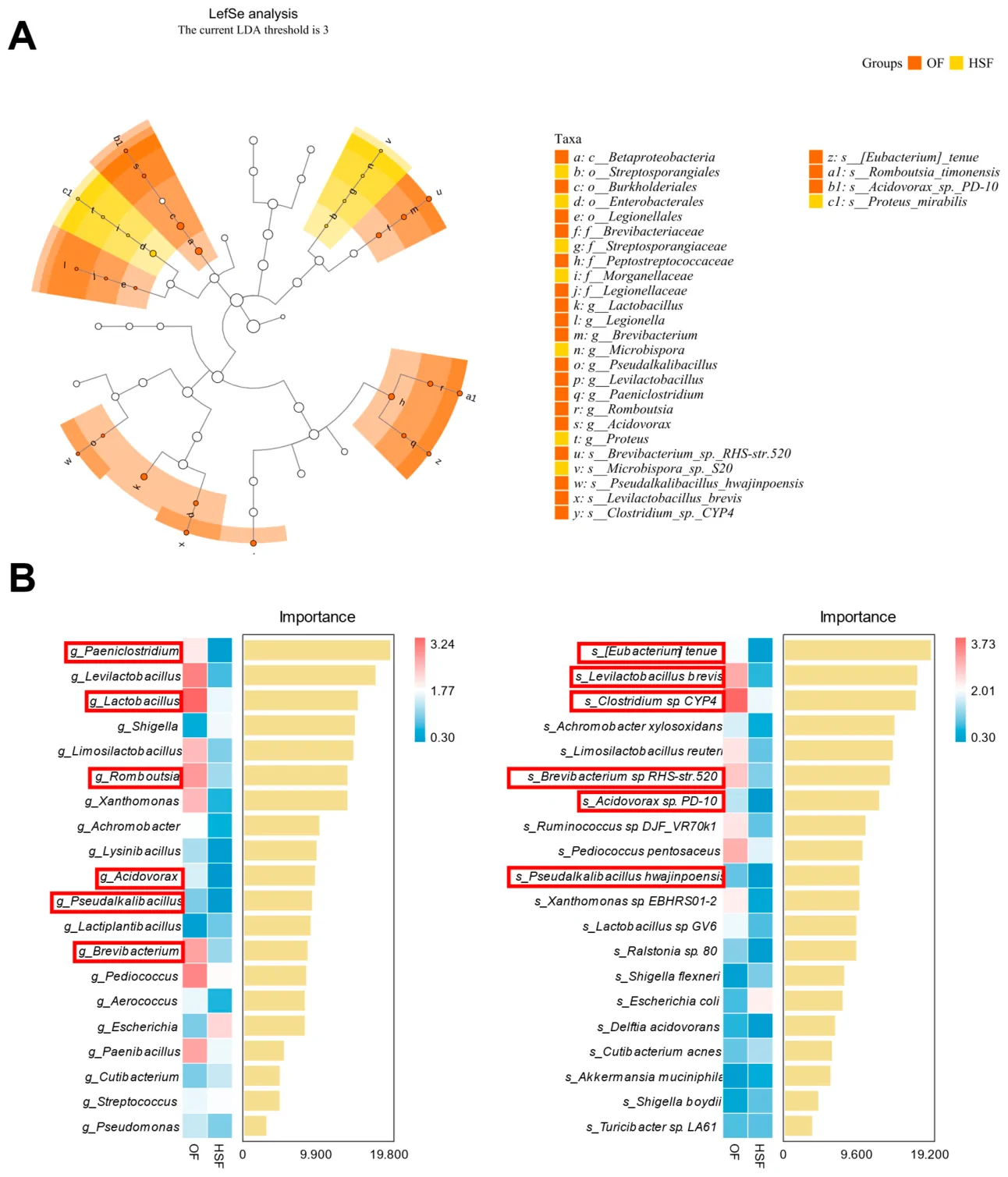
LEfSe analysis and random forest analysis of gut characteristic microbes. LEfSe analysis (**A**); random forest analysis (**B**), The red box contains the strains used in the association analysis.

LEfSe analysis and random forest analysis were employed to identify key differential gut mucosal bacteria between the OF and HSF groups. As shown in Figure 5A,B, 29 characteristic microorganisms were shared across all taxonomic levels among the groups, encompassing 9 species, 10 genera, 5 families, 4 orders, and 1 class. The results of combining LEfSe analysis with random forest analysis show that among the 10 differential genera, 6 have a significant influence value in the random forest, all of which are the dominant genera in the OF group, namely *Paeniclostridium*, *Lactobacillus*, *Romboutsia*, *Acidovorax*, *Pseudalkalibacillus*, and *Brevibacterium*. At the species level, 6 of the 9 differentially abundant species showed high impact values in the random forest model, all being dominant species in the OF group. These were *Eubacterium tenue*, *Levilactobacillus brevis*, *Clostridium* sp. CYP4, *Brevibacterium* sp. RHS-str.520, *Acidovorax* sp. PD-10, and *Pseudalkalibacillus hwajinpoensis*.

### 3.9. Correlation Analysis

#### 3.9.1. Correlation Analysis Between Gut Microbiota and Blood Index

**Figure 6:**
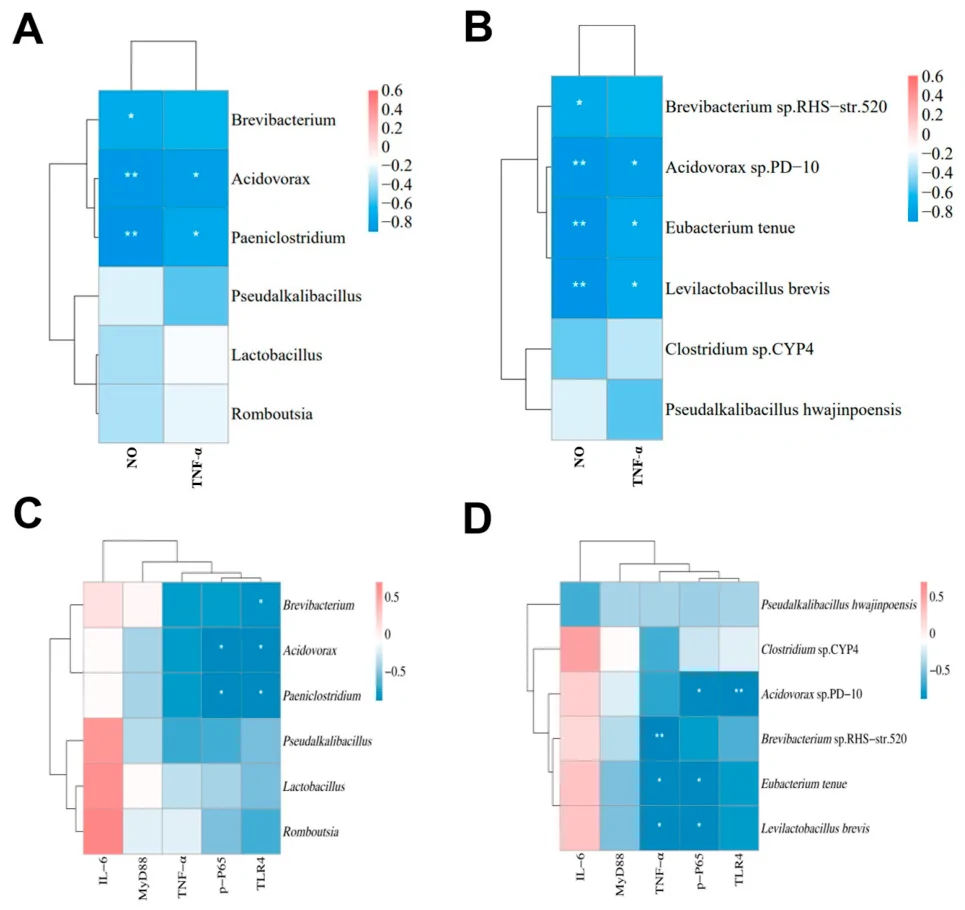
Association Analysis of the Characteristic Gut Microbiota in Rats. Correlation analysis between characteristic gut microbiota and serum index in rats (Left: Genus level; Right: Species level; (**A**,**B**)); correlation analysis between key differential bacteria and protein expression of intestinal MyD88 pathway-related genes (Left: Genus level; Right: Species level; (**C**,**D**)). * indicates *p* ≤ 0.05, ** indicates *p* ≤ 0.01.

As shown in Figure 6A,B, correlation analysis between key differential bacteria and serum NO and TNF-α revealed that at the genus level, *Brevibacterium*, *Acidovorax*, and *Paeniclostridium* showed significant negative correlations with NO (*p* < 0.01, *p* < 0.05), while *Acidovorax* and *Paeniclostridium* exhibited significant negative correlations with TNF-α (*p* < 0.05). At the species level, *Brevibacterium* sp. RHS-str.520, *Acidovorax* sp. PD-10, *Eubacterium* tenue, and *Levilactobacillus brevis* showed significant negative correlations with NO (*p* < 0.01, *p* < 0.05), while *Acidovorax* sp. PD-10, *Eubacterium tenue*, and *Levilactobacillus brevis* showed significant negative correlations with TNF-α (*p* < 0.05). These key differential genera and species were all dominant microbiota in the control group, and the high-salt diet suppressed these key differential microbiota, leading to altered blood NO and TNF-α levels.

#### 3.9.2. Correlation Analysis Between Gut Microbiota and Intestinal Mucosa Protein Expression

As shown in Figure 6C,D, correlation analysis between key differential bacteria and gut MyD88 pathway-related protein expression revealed that *Paeniclostridium* and *Acidovorax* at the genus level showed significant negative correlations with *p*-P65 (*p* < 0.05). *Brevibacterium*, *Paeniclostridium*, and *Acidovorax* exhibited significant negative correlations with TLR4 protein expression (*p* < 0.05). At the species level, *Acidovorax* sp. PD-10, *Eubacterium tenue*, and *Levilactobacillus brevis* showed significant negative correlations with *p*-P65 protein expression (*p* < 0.05). *Brevibacterium* sp. RHS-str.520, *Eubacterium tenue*, and *Levilactobacillus brevis* exhibited negative correlations with TNF-α protein expression (*p* < 0.05, *p* < 0.01), and *Acidovorax* sp. PD-10 showed a significant negative correlation with TLR4 protein expression (*p* < 0.01). Results indicated that *Paeniclostridium* and *Acidovorax* at *the* genus level, and *Acidovorax* sp. PD-10, *Eubacterium tenue*, *Levilactobacillus brevis*, and *Brevibacterium* sp. RHS-str.520 at the species level may be associated with MyD88 pathway activation.

Combining the results of key differential bacteria with serum and intestinal protein expression analysis revealed that the species-level key differential bacteria *Eubacterium tenue* and *Levilactobacillus brevis* were significantly correlated with both intestinal TNF-α protein expression and blood TNF-α levels. These two bacterial species may serve as potential key differential taxa involved in the response to high-salt diet-induced hypertension, and their functional roles warrant further investigation in future studies using approaches such as fecal microbiota transplantation or gnotobiotic animal colonization.

## 4. Analysis and Discussion

Specific environmental factors can disrupt intestinal barrier homeostasis and induce dysbiosis [22]. Long-term high-salt diets can cause damage to the intestinal barrier, leading to bacterial translocation, promoting the entry of intestinal bacterial products into the circulatory system, activating systemic inflammation and inducing hypertension [14,15]. Hypertension also reciprocally affects the gut microbiota, altering its composition and function while impacting intestinal immune function and barrier integrity [23,24]. Our findings indicated that rats developed stable hypertension after 4 weeks on a high-salt diet, accompanied by significantly increased urine output and kidney index during hypertension development. The blood index results showed that the levels of NO and TNF-α in the serum of rats fed a high-salt diet were significantly higher than those of normal rats. The observed increase in serum NO levels in the HSF group might represent a compensatory response to elevated blood pressure. However, as the expression levels of iNOS and eNOS were not directly measured in vascular or intestinal tissues, further studies are required to distinguish whether this reflects chronic inflammation (via iNOS) or an endothelial response (via eNOS). This situation was also supported by the research of YIN Li-tian et al. Their research results indicated that the high expression of iNOS mRNA and protein and the increased iNOS activity induced by a high-salt diet led to the production of a large amount of NO in the myocardial tissue. NO might cause an increase in apoptosis of myocardial cells in salt-sensitive hypertensive rats with sensory nerve damage, thereby aggravating myocardial damage [25]. Urination is closely linked to kidney function. The results of our experiments showed a significant increase in urine volume and a significant increase in the kidney index, indicating that a high-salt diet caused the kidneys of rats to undergo compensatory hyperplasia and hypertrophy, resulting in early pathological changes. Both a high-salt diet and hypertension are risk factors for renal injury. This process involves activation of the renin–angiotensin–aldosterone system, induction of oxidative stress, and triggering of low-grade inflammation. These factors may lead to renal lesions [26].

TLRs serve as pattern recognition receptors that not only play crucial roles in infection immunity and injury-induced inflammatory responses, but also participate in the development of metabolism-related diseases and the regulation of physiological metabolism [27]. Expressed on the cell membrane, TLRs recognize microorganisms through innate and adaptive immune systems, playing a decisive role in the onset and progression of cardiovascular and cerebrovascular diseases [28]. The TLR4/NF-κB pathway mediates multiple chronic diseases [29]. RT-qPCR and WB results from the intestinal TLR4/NF-κB pathways in both rat groups indicated that, compared to the OF group, the relative mRNA expression of MyD88 in the intestinal mucosa of hypertensive rats was significantly increased (*p* < 0.01). The protein expression levels of TLR4, MyD88, *p*-P65, and TNF-α were significantly elevated (*p* < 0.01, *p* < 0.05). Collectively, these multi-omics findings at both gene and protein levels suggest that a high-salt diet interferes with multiple nodes of the intestinal TLR4/NF-κB signaling cascade. At the upstream level, high-salt challenge elevated MyD88 expression, which facilitates signal transduction from the cell-surface receptor to intracellular adaptor molecules; downstream, enhanced phosphorylation of p65 promotes nuclear translocation of NF-κB, driving the transcription of TNF-α pro-inflammatory mediators. TNF-α acts as an early-response pro-inflammatory mediator in this cascade, which can further amplify inflammatory signaling and induce secondary IL-6 production, forming a positive-feedback inflammatory loop within intestinal mucosal tissue. It should be noted that in our present study, only TNF-α exhibited statistically significant elevation at the protein level in intestinal tissue, whereas intestinal IL-6 protein did not reach a significant difference, which is not uncommon in high-salt-induced intestinal inflammation animal models and may be attributed to differences in intervention duration and rat strain-specific inflammatory phenotypes [30]. Combined with the elevated circulating TNF-α observed in peripheral blood, our data suggest that high-salt-induced activation of intestinal TLR4/NF-κB signaling may partially contribute to elevated systemic inflammatory factor levels. Inflammation is a complex self-limiting process serving as a protective mechanism. However, when this self-limiting nature is improperly regulated, it transforms into a harmful chronic inflammatory state, namely chronic low-grade inflammation. This chronic low-grade inflammatory process is essential for the development of hypertension [31,32,33]. Combined with the results of intestinal mechanical barrier and immunofluorescence staining, it can be seen that a high-salt diet results in a significant decrease in EL, and a downward trend in the expression of Claudin-1 and ZO-1 proteins, as well as autolysis at the tip of the villi and incomplete mucosal structure. Therefore, a high-salt diet may exert a potential impact on the mechanical barrier of the rat intestine. Taken together, these findings suggest that a high-salt diet may damage intestinal barrier integrity, which in turn may activate intestinal pattern recognition receptors and alter the protein expression of downstream inflammatory factors, thereby contributing to elevated circulating inflammatory cytokine levels and maintaining the body in a prolonged low-grade inflammatory state, potentially participating in the development of hypertension.

Results for dominant and key differential microorganisms at the genus and species levels in the intestinal mucosa indicate that a high-salt diet significantly suppressed beneficial bacteria at both levels, including genera such as *Clostridium*, *Lactobacillus*, *Acidovorax*, and *Brevibacterium*, as well as species like *Ligilactobacillus*, *Eubacterium tenue*, and *Levilactobacillus brevis*, while promoting the growth of the harmful genus *Nodosilinea* [20]. The genus *Clostridium* is mostly non-pathogenic. Most bacteria in this genus can hydrolyze sugars and proteins. For example, *Clostridium butyricum* promotes the growth of beneficial bacteria (*Bifidobacterium* and *Lactobacillus*) while inhibiting pathogenic bacteria, thereby regulating intestinal health [34,35]. In our experiment, the relative abundance of *Clostridium* in the OF group was higher than that in the HSF group. *Lactobacillus* are probiotics in the gut. They produce short-chain fatty acids and gamma-aminobutyric acid in the intestine, promoting gut health and lowering blood pressure. Producing GABA-producing *Lactobacillus* may be beneficial for treating intestinal dysfunction [36,37]. *Ligilactobacillus* is a subgenus of *Lactobacillus*. As a beneficial bacterium, it can stimulate immune cells to secrete anti-allergy-related cytokine concentrations [38,39]. Gut microbiota results of our experiment indicate that high-salt diets damage the intestinal barrier, leading to bacterial dysbiosis. This inhibits beneficial bacteria growth while promoting harmful bacteria proliferation. Analysis of key differential bacteria correlated with blood index and downstream protein expression in the TLR4/NF-κB pathway revealed that the species-level *Brevibacterium* sp. RHS-str.520 and *Levilactobacillus brevis* from the OF group showed significant negative correlations with serum TNF-α and TNF-α protein expression in the MyD88 pathway, suggesting these two bacteria may be associated with TNF-α production in high-salt diet-induced hypertension. At the species level, *Acidovorax* sp. PD-10, *Eubacterium tenue*, *Levilactobacillus brevis*, and *Brevibacterium* sp. RHS-str.520 may be associated with MyD88 pathway activation. Majid et al. Have demonstrated that a high-salt diet can induce TNF-α production, which activates its receptor type 1 to exert a natriuretic effect [40]. Our results suggest that this effect may be associated with high-salt-diet-mediated suppression of *Brevibacterium* sp. RHS-str.520 and *Levilactobacillus brevis*. This may activate the TLR4/NF-κB pathway and up-regulate downstream protein levels of TLR4, MyD88, *p*-P65, and TNF-α, which may further elevate serum TNF-α, potentially contributing to chronic low-grade inflammation and the development of hypertension.

## 5. Conclusions

In summary, a high-salt diet may damage the intestinal mucosal barrier of rats, as evidenced by villus tip autolysis, epithelial cell detachment, decreased villus height, significant shortening of epithelial length, and reduced crypt depth, which may lead to increased intestinal permeability. Meanwhile, a high-salt diet suppresses the growth of key differential bacteria *Brevibacterium* sp. RHS-str.520 and *Levilactobacillus brevis*. This may enable pathogenic bacteria to translocate across a damaged intestinal barrier and be sensed by the TLR4 receptor. Such recognition may trigger the TLR4/NF-κB inflammatory cascade, which could contribute to the release of inflammatory cytokines. These alterations may contribute to chronic low-grade inflammation in rats, which could be associated with the development of hypertension. Collectively, these mechanistic interpretations are inferred from correlative analyses of our present findings. Direct experimental evidence is still required in future work.

### Limitations

This study has several limitations. First, this study is observational in nature, and no pathway-specific inhibitors or gene knockout models were employed for mechanistic validation. Future studies using pharmacological interventions or genetic approaches are warranted to further clarify the direct regulatory mechanisms. Second, this study did not measure gut microbial metabolites (e.g., short-chain fatty acids). Future investigations may integrate metabolomic profiling with gut microbiota data to provide a more complete mechanistic understanding. Third, the relatively brief experimental period and modest sample size in our work may partially limit the robustness and broader applicability of the present findings. Future research incorporating longer-term monitoring and larger cohorts would help further corroborate the findings reported here. Fourth, the sample size was relatively small (six animals per group), limiting statistical power; therefore, multiple-testing correction was not applied, and all differential taxa and correlation findings should be regarded as exploratory. Future studies with larger sample sizes and more rigorous statistical approaches are warranted.

## Figures and Tables

**Table 1:** Composition of ordinary feed and high-salt feed.

Constituent Content	Ordinary Feed	High-Salt Feed
Water	<10%	<10%
Crude Protein	≥18%	≥18%
Crude Fat	≥4%	≥4%
Crude Fiber	≤5%	≤5%
Coarse ash	≤8%	≤8%
Ca	1.0–1.8%	1.0–1.8%
P	0.6–1.2%	0.6–1.2%
NaCl	1%	8%

**Table 2:** Effects of high-salt diet on rat blood pressure (*n* = 6).

Time (Day)	Blood Pressure	OF (mmHg)	HSF (mmHg)
2	SBP	121.36 ± 7.52	114.81 ± 13.42
	DBP	92.36 ± 8.66	92.49 ± 14.06
5	SBP	118 ± 6.03	128.71 ± 10.86 *
	DBP	92.07 ± 7.13	99.89 ± 6.99 *
9	SBP	115 ± 7.06	124.1 ± 6.99 **
	DBP	88.83 ± 5.14	95.37 ± 8.86
12	SBP	113.56 ± 13.79	119.34 ± 5.88
	DBP	90.24 ± 13.93	93.43 ± 10.22
16	SBP	115.53 ± 11.09	135.51 ± 11.44 **
	DBP	92.4 ± 8.29	107.48 ± 7.64
19	SBP	122.2 ± 9.10	128.72 ± 7.72
	DBP	89.91 ± 4.78	97.53 ± 10.38 *
23	SBP	123.14 ± 8.80	127.93 ± 12.31
	DBP	95.07 ± 6.71	99.48 ± 14.84
26	SBP	121.4 ± 7.58	147.26 ± 15.01 **
	DBP	96.04 ± 6.13	115 ± 11.16 **
30	SBP	114.42 ± 11.13	142.13 ± 13.55 **
	DBP	90.64 ± 13.19	112.21 ± 11.39 **
33	SBP	120.2 ± 6.01	147.3 ± 9.43 **
	DBP	94.13 ± 9.09	118.26 ± 14.06 **

Note: Compared with the OF group, * indicates *p* ≤ 0.05, ** indicates *p* ≤ 0.01.

**Table 3:** Comparison of intestinal VH, CD, V/C ratio, goblet cell count, and EL among rat groups (*n* = 6).

Group	VH/µm	CD/µm	VH/CD	Goblet Cells	EL/µm	Goblet Cells per Unit Length/mm
OF	379.68 ± 0.04	216.32 ± 0.03	1.76 ± 0.07	16.67 ± 5.55	663.72 ± 0.04	25.38 ± 9.22
HSF	364.17 ± 0.06	199.74 ± 0.01	1.83 ± 0.33	15.87 ± 3.52	509.00 ± 0.05 *	30.94 ± 4.21

Note: Compared with the OF group, * indicates *p* ≤ 0.05.

## Data Availability

The original contributions presented in this study are included in the article. Further inquiries can be directed to the corresponding author.
